# Bismuth Drugs Reverse Tet(X)-Conferred Tigecycline Resistance in Gram-Negative Bacteria

**DOI:** 10.1128/spectrum.01578-21

**Published:** 2022-02-09

**Authors:** Tian Deng, Yuqian Jia, Ziwen Tong, Jingru Shi, Zhiqiang Wang, Yuan Liu

**Affiliations:** a College of Veterinary Medicine, Yangzhou University, Yangzhou, Jiangsu, China; b Jiangsu Co-innovation Center for Prevention and Control of Important Animal Infectious Diseases and Zoonoses, Joint International Research Laboratory of Agriculture and Agri-Product Safety, the Ministry of Education of China, Yangzhou University, Yangzhou, Jiangsu, China; c Institute of Comparative Medicine, Yangzhou University, Yangzhou, Jiangsu, China; Hartford Hospital

**Keywords:** antibiotic adjuvant, bismuth drugs, *tet*(X), Gram-negative bacteria

## Abstract

Antibiotic resistance has caused a serious threat to public health and human safety. Recently, the emergence of novel resistance gene *tet*(X4) and its variants threatens the clinical utility of tigecycline, one of the last-line antibiotics for multidrug-resistant (MDR) bacterial infections. It is highly promising to develop effective antibiotic adjuvants to restore the clinical efficacy of existing drugs and extend their life spans. Metal compounds, such as silver, have been widely used as potential antimicrobial agents for decades. However, the potentiating effect of metallo-agents on the existing antibiotics is not fully understood. Here, we found that five bismuth drugs, especially bismuth nitrate [Bi(NO_3_)_3_], commonly used in clinical treatment of stomach-associated diseases, effectively boost the antibacterial activity of tigecycline against *tet*(X)-positive bacteria by inhibiting the enzymatic activity of Tet(X) protein. Furthermore, the combination of Bi(NO_3_)_3_ and tigecycline prevents the development of higher-level resistance in Tet(X)-expressing Gram-negative bacteria. Using molecular docking and dynamics simulation assays, we revealed that Bi(NO_3_)_3_ can competitively bind to the active center of Tet(X4) protein, while the bismuth atom targets the Tet(X4) protein in a noncompetitive manner and changes the structure of the primary binding pocket. These two mechanisms of action both antagonize the enzymatic activity of Tet(X4) resistance protein on tigecycline. Collectively, these findings indicate the high potential of bismuth drugs as novel Tet(X) inhibitors to treat *tet*(X4)-positive bacteria-associated infections in combination with tigecycline.

**IMPORTANCE** Recently, high-level tigecycline resistance mediated by *tet*(X4) and its variants represents a serious challenge for global public health. Antibiotic adjuvant strategy that enhances the activity of the existing antibiotics by using nonantibiotic drugs offers a distinct approach to combat the antibiotic resistance crisis. In this study, we found that bismuth drugs involve bismuth nitrate, a compound previously approved for treatment of stomach-associated diseases, remarkably potentiates tigecycline activity against *tet*(X)-positive bacteria. Mechanistic studies showed that bismuth drugs effectively suppress the enzymatic activity of Tet(X) resistance protein. Specifically, bismuth nitrate targets the active center of Tet(X4) protein, while bismuth binds to the resistance protein in a noncompetitive manner. Our data open up a new horizon for the treatment of infections caused by *tet*(X)-bearing superbugs.

## INTRODUCTION

Tigecycline belongs to the third generation of tetracycline antibiotics with broad-spectrum antibacterial activity (1). The World Health Organization lists it as an extremely important antibacterial drug for the treatment of clinical multidrug-resistant (MDR) bacterial infections (2). Under the current medical clinical situation, in which Gram-negative bacteria are increasingly resistant to carbapenems, tigecycline and polymyxin have become two of the few options for the treatment of MDR bacterial infections. In recent years, as the mobilized colistin resistance (MCR)-mediated transferable polymyxin resistance mechanism was identified (3), tigecycline has been recognized as the last line of defense currently known to combat these “superbugs.” However, high-level tigecycline resistance genes *tet*(X3) and *tet*(X4) were discovered in 2019 in humans and animals (4). The prevalence of plasmid-mediated *tet*(X3/X4) genes has seriously threatened its clinical application in the treatment of bacterial infectious diseases, and it can be transferred into Escherichia coli, Acinetobacter baumannii, Klebsiella pneumoniae, and other clinically important pathogens through conjugation (5). Thus, new strategies are urgently needed to deal with the increasing threat of Tet(X)-conferred tigecycline resistance.

Alternatively, compared to the novel antibiotic discovery, antibiotic adjuvant therapies, such as the inhibitors of resistance enzyme, provide a more cost-effective strategy to tackle the resistance crisis (6–8). The β-lactamase inhibitor, widely used in clinical practice, is a typical example. It can inhibit the activity of enzyme by forming a stable complex with β-lactamase, thereby displaying a synergistic effect with β-lactam antibiotics (9). Since 1969, through microbial screening and structural modification of enzyme inhibitors, a variety of β-lactamase inhibitors have been obtained, such as clavulanic acid, sulbactam, and tazobactam (10). Some metal compounds have excellent antimicrobial activity by damaging protein, DNA, and other biological macromolecules in bacteria (11), such as silver (12), copper (13), etc. In response to the increasingly serious multidrug resistance crisis, whether metal compounds are able to play a potential role in restoring the activity of existing antibiotics has aroused great interest. For example, Morones-Ramirez et al. showed that silver potentiated the antibiotics activity against Gram-negative bacteria in different metabolic states by disrupting multiple bacterial cellular processes, triggering the enhanced reactive oxygen species (ROS) production and bacterial membrane permeability (14). Auranofin, an antirheumatic drug, is a dual inhibitor of metallo-β-lactamases (MBLs) and mobilized colistin resistance enzymes (MCRs) via the displacement of Zn (II) cofactors from their active sites (15). Bismuth compounds are drugs clinically approved for treating Helicobacter pylori-associated infections, and no resistance was found over past decades (16). Additionally, bismuth compounds exhibit many interesting properties that have led to numerous applications ranging from organic synthesis to engineering (17, 18). Therefore, repurposing metal drugs as potential antibiotic adjuvants represents a promising alternative to tackle the current antimicrobial resistance crisis.

In this study, by using checkerboard assays, we systematically assessed the synergistic activity between Bi(NO_3_)_3_, a drug used clinically to treat peptic ulcer, diarrhea, and enteritis (19), and different classes of antibiotic against MDR Gram-negative bacteria. As a result, we found that Bi(NO_3_)_3_ remarkably restores the activity of meropenem and tigecycline against New Delhi metallo-β-lactamase (NDM)-positive and Tet(X4)-positive bacteria, respectively. Furthermore, we showed that Bi(NO_3_)_3_ significantly prevents the evolution of Tet(X) by mutant prevention concentration (MPC) analysis and resistance development studies. Finally, we explored the underlying modes of action of Bi(NO_3_)_3_ and Bi atom in antagonizing the enzymatic activity of Tet(X) protein via molecular docking and dynamics simulation analysis. Our results strongly suggest that Bi(III) drugs or compounds may serve as the novel inhibitors of Tet(X) resistance enzymes and provide a promising combination therapy for the treatment of infections caused by Tet(X)-positive pathogens.

## RESULTS

### Bismuth drugs have excellent synergistic activity with tigecycline.

**FIG 1:**
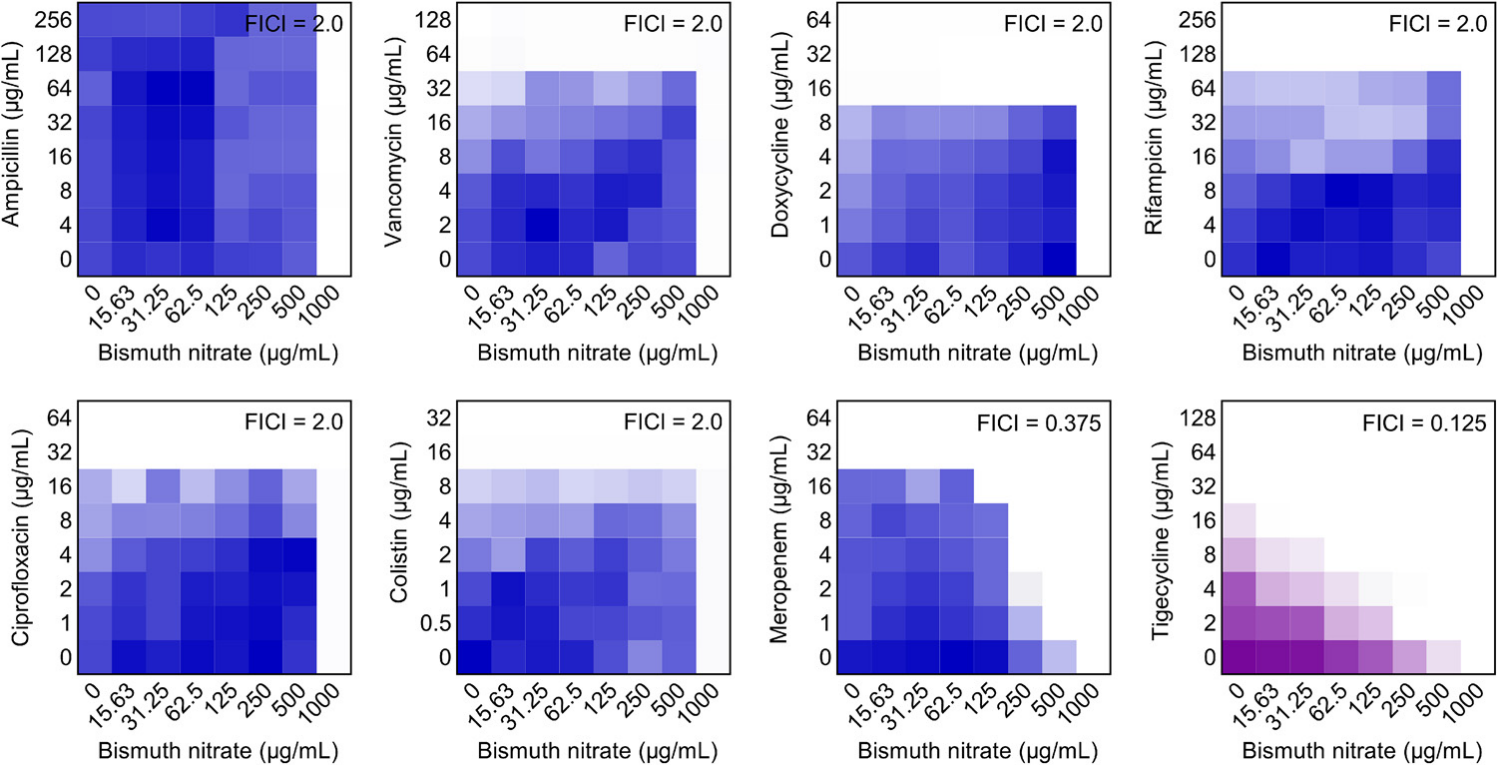
Synergistic activity of bismuth nitrate and antibiotics against MDR E. coli. Checkerboard assay between bismuth nitrate and various antibiotics against MDR E. coli B2 coharboring *bla*_NDM-5_ and *mcr-1* or tigecycline-resistant E. coli B3-1 carrying *tet*(X4). After the bacterial suspension was added, the flat-bottom plate was cultured at 37°C for 16 to 18 h, and then the absorbance at 600 nm was determined. In the heat map, the darker the color, the greater the bacteria density. The data represent the averages of two biological replicates.

To evaluate the synergistic effect between bismuth nitrate [Bi(NO_3_)_3_] and eight antibiotics with different mechanisms of action, we performed checkerboard broth microdilution assays using two MDR isolates, E. coli B2 coharboring *bla*_NDM-5_ and *mcr-1* or E. coli B3-1 carrying *tet*(X4), as tested strains. Tigecycline-resistant E. coli B3-1 was used only for evaluating the drug-drug interaction between Bi(NO_3_)_3_ and tigecycline. Interestingly, we found that Bi(NO_3_)_3_ merely displayed a synergistic effect with meropenem against E. coli B2 with a fractional inhibitory concentration index (FICI) value of 0.375 (Fig. 1 and Table S1). Consistent with our observation, a recent study indicated the high potential of Bi(III) compounds, such as colloidal bismuth subcitrate (CBS), as the first broad-spectrum B1 MBL inhibitors by displacing Zn(II) ions and leading to the release of Zn(II) cofactor (20). Notably, we unexpectedly found that Bi(NO_3_)_3_ displayed better potentiation to tigecycline (FICI = 0.125) against *tet*(X4)-positive E. coli B3-1 (Fig. 1 and Table S1). Given the high priority and important role of tigecycline in treatment of serious infections in clinical practice, we focused our following studies on the synergy potency of Bi(NO_3_)_3_ with tigecycline in the fight against drug-resistant pathogens.

**FIG 2:**
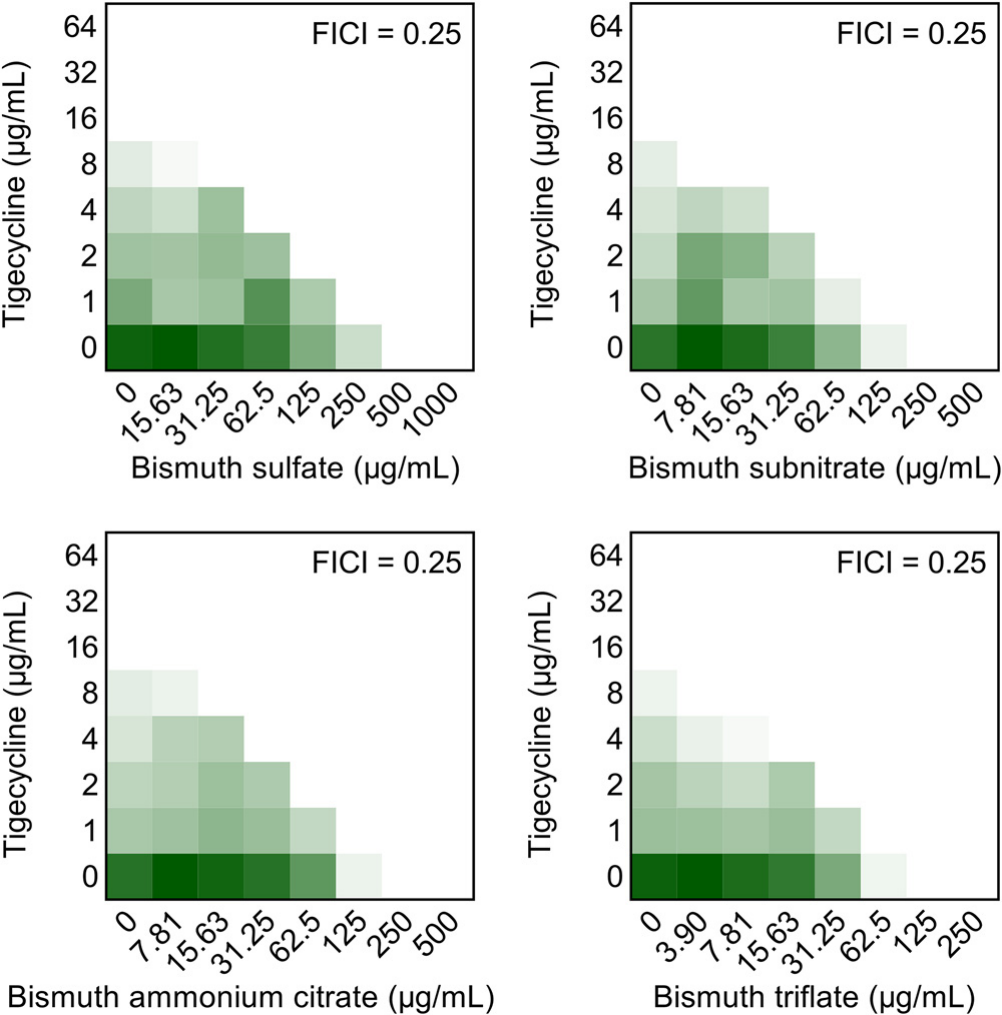
Four bismuth drugs, including bismuth sulfate, bismuth subnitrate, bismuth ammonium citrate, and bismuth triflate, effectively potentiate tigecycline activity against engineered E. coli DH5α [PMD19-*tet*(X4)]. Dark green regions represent higher cell density. The data represent the average of two biological replicates.

We first assessed the synergistic effect of Bi(NO_3_)_3_ with tigecycline on a panel of *tet*(X4)-positive clinical pathogens from different sources (21). The MIC values of tigecycline were remarkably reduced by 4- to 16-fold in the presence of a quarter-MIC of Bi(NO_3_)_3_, accompanied by FICI values ranging from 0.125 to 0.375 (Table S2). Next, we determined whether other bismuth drugs can sensitize tigecycline against an engineered *tet*(X4)-positive strain, E. coli DH5α [PUC19-*tet*(X4)]. The results of checkerboard assays showed that all four Bi(III) compounds, including bismuth sulfate, bismuth subnitrate, bismuth ammonium citrate, and bismuth triflate, remarkably synergized with tigecycline (FICI = 0.25) in *tet*(X4)-bearing bacteria (Fig. 2 and Table S3). These results imply that bismuth drugs are potential tigecycline adjuvants in the fight against *tet*(X4)-positive pathogens.

### Bi(NO_3_)_3_ suppresses resistance evolution of tigecycline.

**FIG 3:**
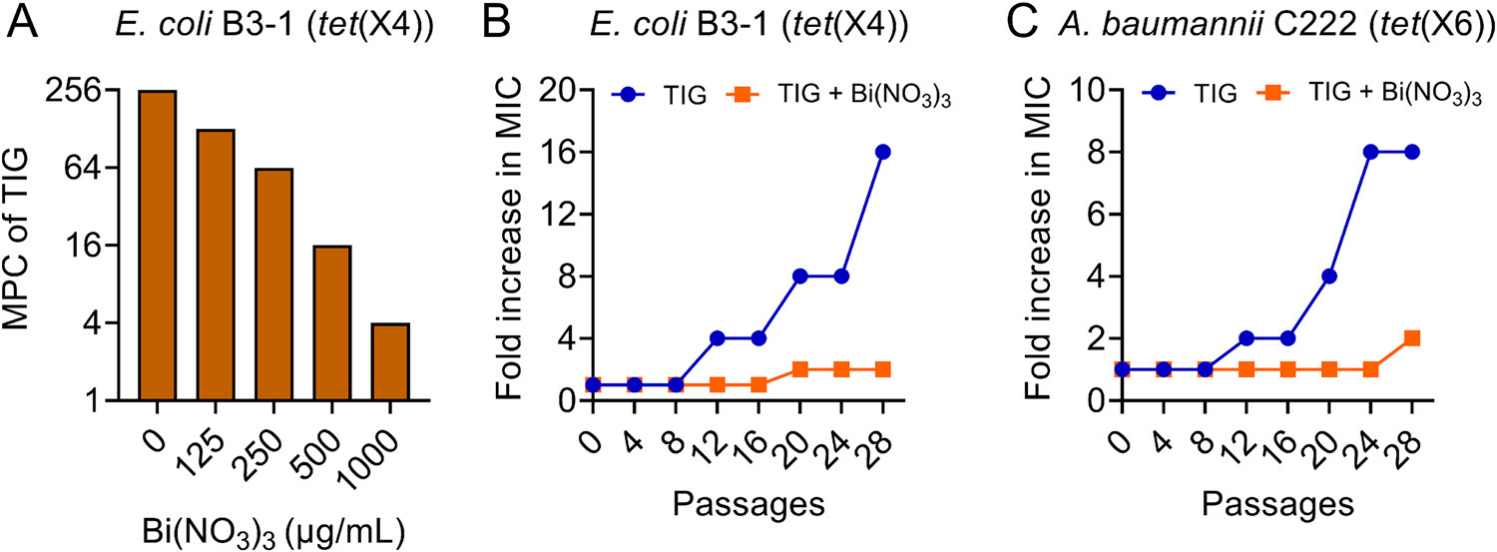
Bismuth nitrate suppresses the evolution of tigecycline resistance. (A) MPC values of tigecycline against E. coli B3-1 in the presence of different concentrations of bismuth nitrate ranging from 0 to 1,000 μg/mL. (B and C) Serial passages of two *tet*(X)-positive isolates at subinhibitory concentrations of tigecycline or the combination of tigecycline and bismuth nitrate. The MIC values were measured at every four passages.

Although bismuth drugs have been widely used for the clinical treatment of H. pylori-related diseases for decades (22), no obvious resistance was reported. This may be attributed to the fact that metal compounds can target multiple bacterial biological pathways (18, 23). Considering the great potentiation of Bi(NO_3_)_3_ to tigecycline, we next determined whether its unique characteristics have the potency to prevent the development of resistance by MPC and resistance development studies. MPC was determined for tigecycline in the absence and presence of different concentrations of Bi(NO_3_)_3_. We found that the addition of Bi(NO_3_)_3_ substantially reduced the MPC values of tigecycline in *tet*(X4)-positive bacteria in a concentration-dependent manner (Fig. 3A). The MPC of tigecycline was lowered to 4 μg/mL with a 64-fold reduction when the concentration of Bi(NO_3_)_3_ increased to 1,000 μg/mL. Then, over a period of 28 consecutive passages of two clinical tigecycline-resistant bacteria, namely, E. coli B3-1 (Fig. 3B) and A. baumannii C222 (Fig. 3C), upon exposure to subinhibitory concentrations of tigecycline, resistance levels to tigecycline were greatly elevated by 16- and 8-fold, respectively. In contrast, the combined use of Bi(NO_3_)_3_ and tigecycline significantly slowed the increase of MIC values in two *tet*(X)-positive pathogens, with only 2-fold alteration. Collectively, these results indicate that Bi(NO_3_)_3_ can significantly suppress the evolution of Tet(X) enzyme and the development of high-level tigecycline resistance.

### Bi(NO_3_)_3_ inhibits the enzymatic activity of Tet(X4) protein.

**FIG 4:**
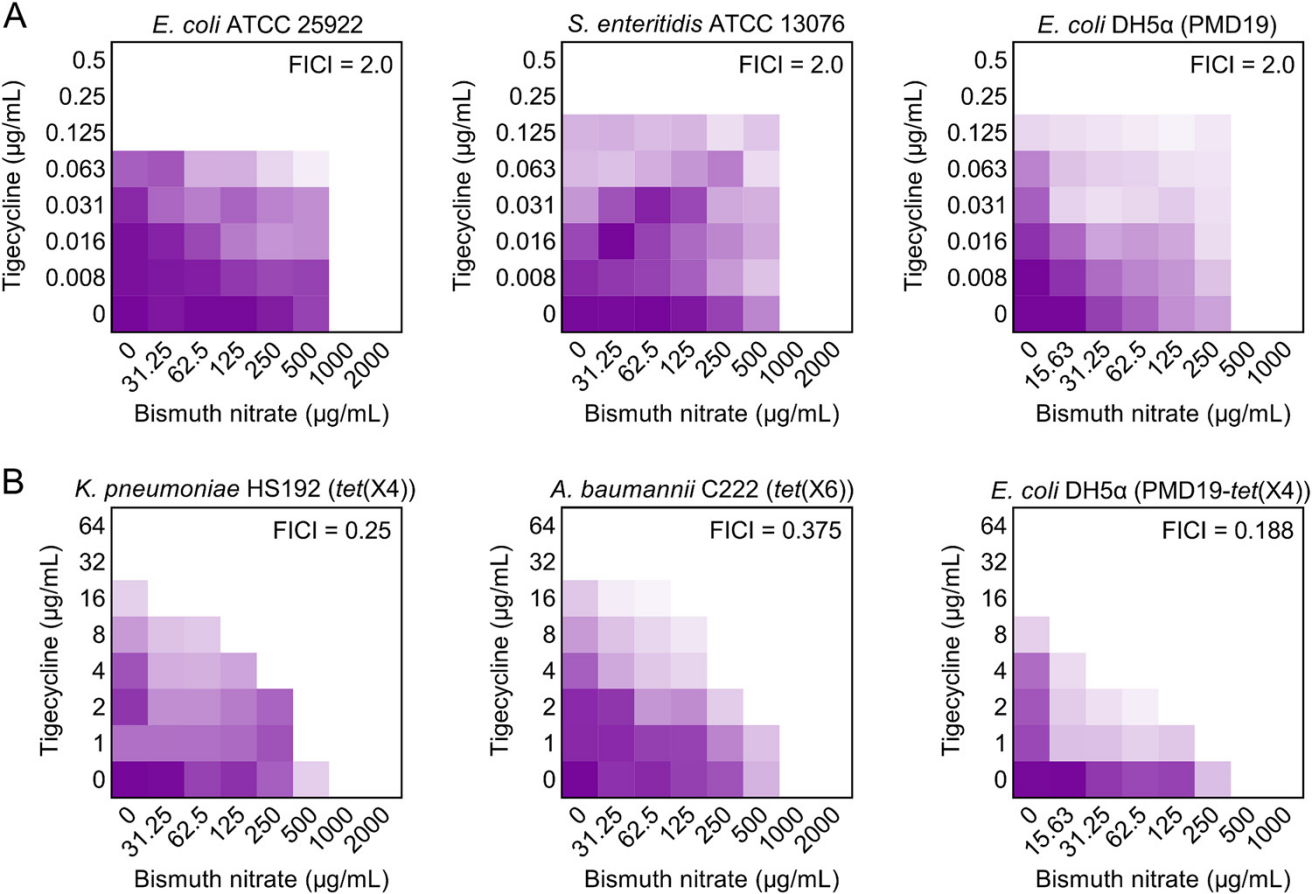
Bismuth nitrate remarkably enhances tigecycline activity in *tet*(X)-positive bacteria, not in *tet*(X)-negative bacteria. Checkerboard assay between bismuth nitrate and tigecycline against *tet*(X)-negative bacteria (A) or *tet*(X)-positive bacteria (B). Dark purple regions represent higher cell density. The data represents the average of two biological replicates.

**FIG 5:**
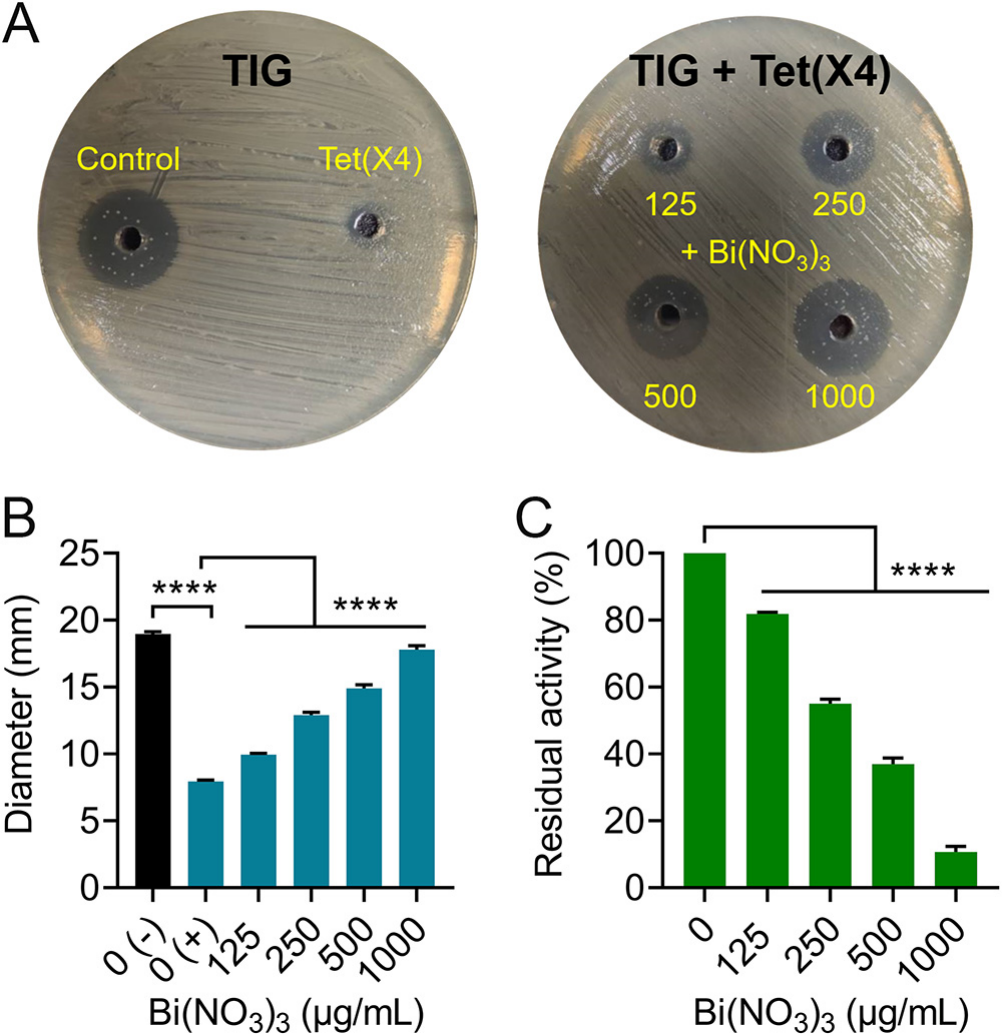
Bismuth nitrate effectively inhibits the activity of Tet(X4) enzyme. (A) The representative inhibition zone of tigecycline after Tet(X4) incubation (left) and the expansion of the inhibition zones under the action of bismuth nitrate (right). (B and C) The changes of inhibition zones (B) and normalized residual activity (C) of tigecycline after coincubation with Tet(X4) containing supernatants alone or in combination with different concentrations of bismuth nitrate. 0 (−): supernatants without treatment added; 0 (+): supernatants treated with Tet(X4) added. The data are representative of three biological replicates and expressed as mean ± standard deviation (SD). Significant differences were analyzed by one-way analysis of variance (ANOVA) and shown with ****, *P < *0.0001.

To elucidate the mechanisms of action underlying the potentiation of Bi(NO_3_)_3_ to the antimicrobial activity of tigecycline against *tet*(X)-bearing bacteria, we determined the synergistic activity of Bi(NO_3_)_3_ and tigecycline in three *tet*(X4)-negative bacteria and three *tet*(X4)-positive bacteria, which include clinical and engineered bacteria to exclude the impact of strains’ background differences. Consistently, these *tet*(X4)-positive bacteria conferred resistance to tigecycline with MIC values from 16 to 32 μg/mL, whereas *tet*(X4)-negative bacteria were sensitive to tigecycline. Interestingly, Bi(NO_3_)_3_ had a synergistic activity with tigecycline in *tet*(X4)-positive bacteria only with FICI values from 0.188 to 0.375, while such synergism was not observed in tigecycline-sensitive bacteria (FICI = 2.0) (Fig. 4 and Table S4). Meanwhile, the synergy potency of Bi(NO_3_)_3_ with minocycline in three *tet*(X)-positive bacteria was also observed (Fig. S1), because *tet*(X) also confers resistance to minocycline. These results implied that Bi(NO_3_)_3_ may specifically inhibit the enzymatic activity of Tet(X4). To verify this hypothesis, we conducted Tet(X) enzyme-degrading assays under exposure of Bi(NO_3_)_3_. Tet(X4) enzyme was expressed by constructing the expression vector BL21(DE3) + pBAD30-*tet*(X4). As shown in Fig. 5A and B, compared with that of the untreated group, the inhibition zone of tigecycline against E. coli ATCC 25922 after coculture with Tet(X4) enzyme was markedly decreased, indicating that the activity of tigecycline was abolished by Tet(X4) resistance enzyme. Importantly, in the presence of Bi(NO_3_)_3_, the inhibition zone of tigecycline was dose-dependently increased despite the effect of Tet(X4) enzyme, implying the recovery of tigecycline activity and the inhibitory effect of Bi(NO_3_)_3_ on Tet(X4) protein. The residual of Tet(X) enzymes in the absence of Bi(NO_3_)_3_ was set as 100%; we found that the enzymatic activity of Tet(X) was remarkably decreased as the concentrations of Bi(NO_3_)_3_ increased (Fig. 5C). Specifically, the activity of Tet(X) resistance enzyme was lower than 10% after treatment with 1,000 μg/mL Bi(NO_3_)_3_. To further verify whether the synergy of Bi(NO_3_)_3_ is related only to *tet*(X)-mediated tigecycline resistance, we tested the synergistic activity between bismuth nitrate and tigecycline against *tmexCD1-toprJ1*- or *tet*(A) variant-mediated tigecycline-resistant bacteria (24, 25). However, no direct synergistic activity (FICI values of >0.5) was found in these strains (Fig. S2). These data demonstrate that Bi(NO_3_)_3_ effectively inactivates Tet(X) resistance enzyme, thereby restoring tigecycline activity against *tet*(X)-positive pathogens.

### Bi(NO_3_)_3_ has a higher affinity with the active center of Tet(X4) protein.

**FIG 6:**
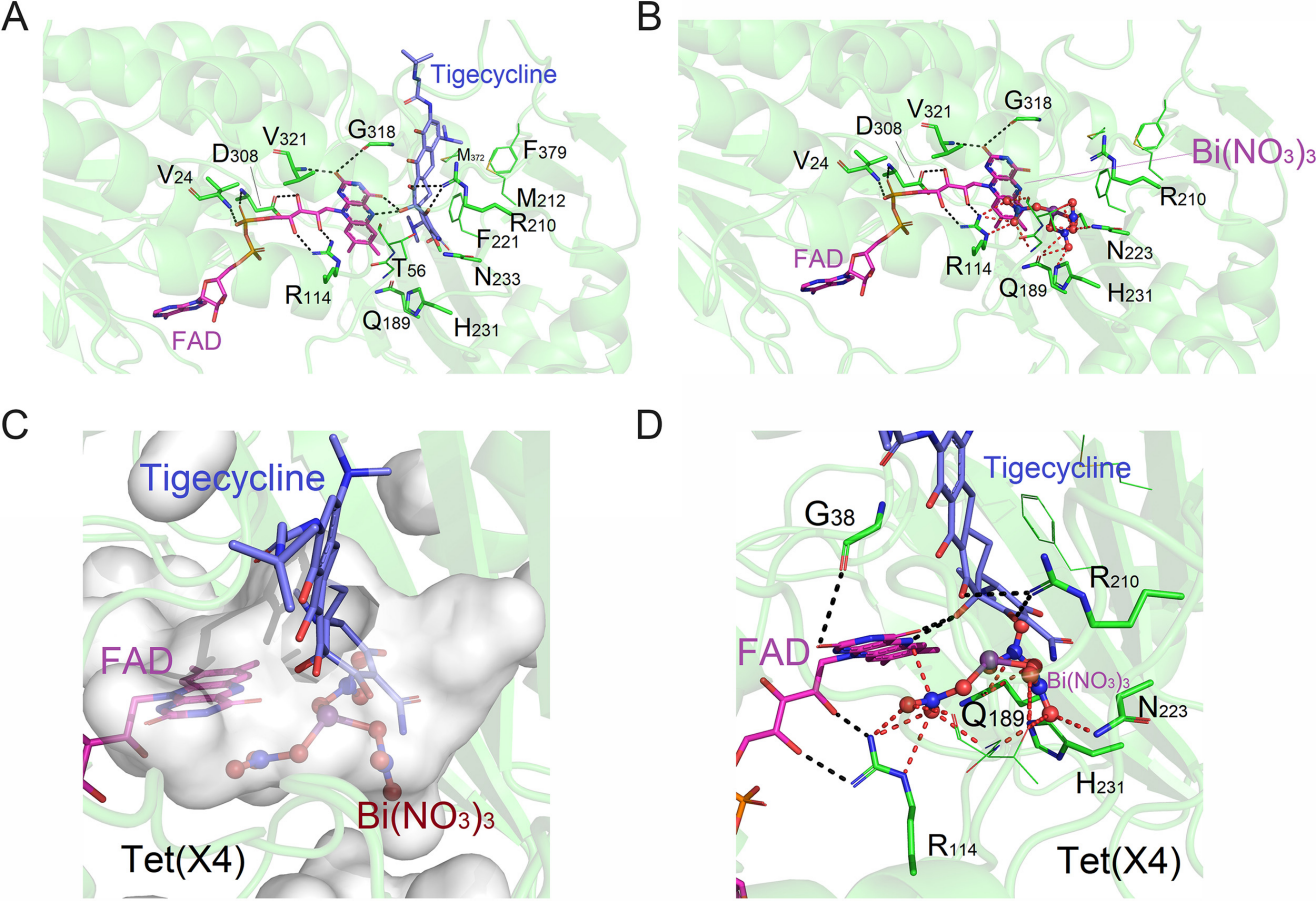
Molecular docking analysis of Tet(X4) with FAD and tigecycline (A) or bismuth nitrate (B), respectively. The binding energies are −6.29 kcal/mol and −8.42 kcal/mol, respectively. (C and D) Overlay diagrams of Tet(X4) protein cavity-Bi(NO_3_)_3_-tigecycline structure.

To understand how Bi(NO_3_)_3_ inhibits the activity of Tet(X4) enzyme from a molecular perspective, we applied molecular docking to compare the interaction of two drugs with Tet(X4) enzyme, respectively. Interestingly, we found that both Bi(NO_3_)_3_ and tigecycline could bind to the active center of Tet(X4) protein, with the binding energies of −8.42 and −6.29 kcal/mol, respectively (Fig. 6A and B). The lower binding energy between Bi(NO_3_)_3_ and Tet(X4) protein indicated that Bi(NO_3_)_3_ had a higher affinity with the active center of Tet(X4) protein. Specifically, Bi(NO_3_)_3_ occupied the binding cavity after entering the active center and formed a strong polar interaction with the Tet(X4) protein and flavin adenine dinucleotide (FAD) molecules (Fig. 6C and D). Tigecycline could not compensate for the energy formed by the Bi(NO_3_)_3_-Tet(X4) conjugate, and thus it was at a disadvantage in the competitive antagonism. These data indicate that Bi(NO_3_)_3_ can competitively bind to the active center of Tet(X4) proteins and prevent its enzymatic effect on tigecycline, thereby boosting its antibacterial activity.

### Bismuth prevents the binding between Tet(X4) protein and tigecycline.

**FIG 7:**
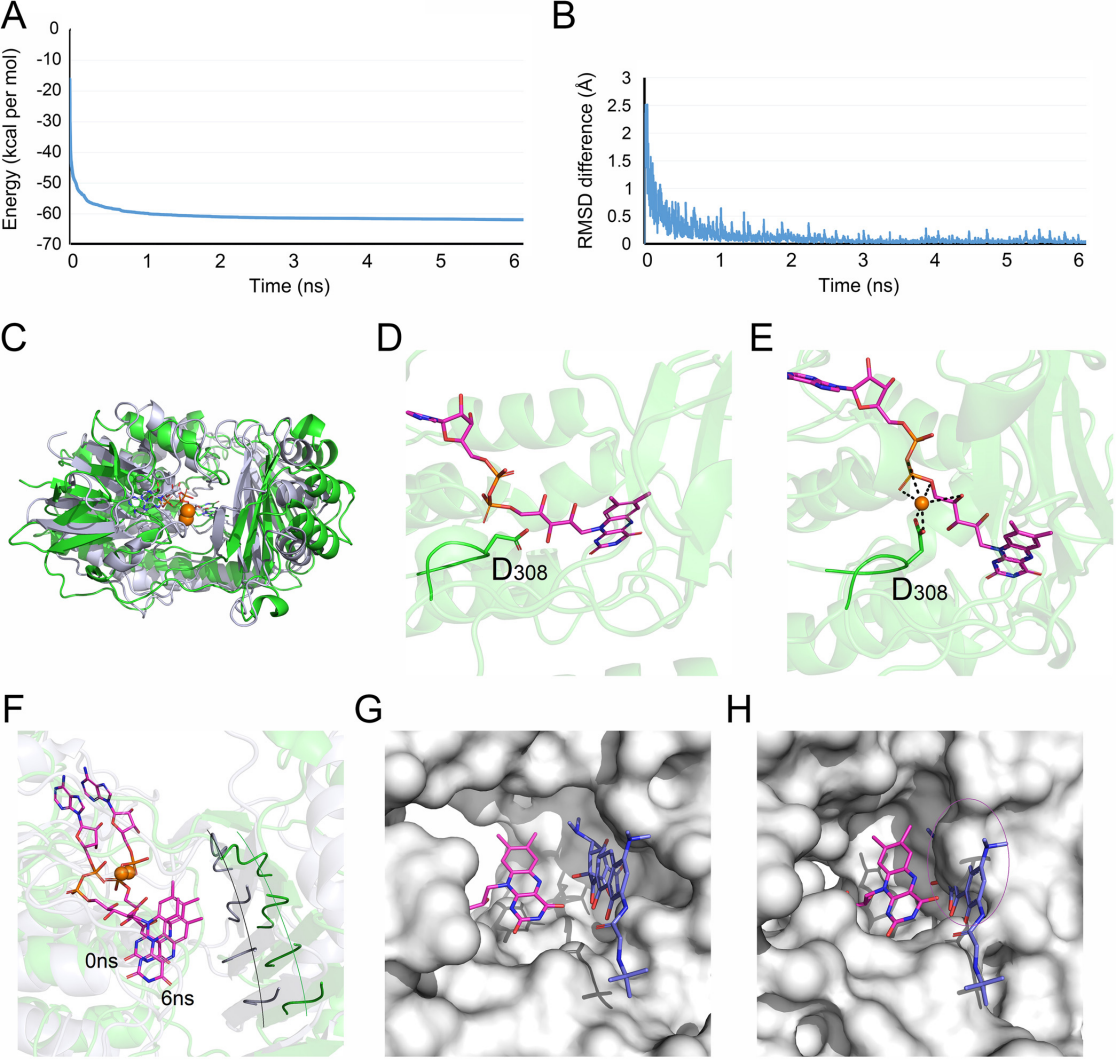
Dynamics simulation analysis of Tet(X4) protein with FAD and bismuth atom. (A and B) Protein energy (A) and RMSD differentiation (B) change over 6 ns in the presence of bismuth atom. (C) Overlay diagram of the 0 ns and 6 ns protein complex structures. Green: 0 ns structure; white: 6 ns structure. (D and E) The binding diagrams of D308 in the absence (D) or presence (E) of bismuth atoms. (F) Structural change of molecule pocket binding sites at 0 ns and 6 ns. Protein binding pocket was expressed by ribbon; green: 0 ns; white: 6 ns. (G and H) Protein binding pockets at 0 (G) and 6 ns (H). In the presence of bismuth, the reduced space in the binding pocket prevents tigecycline molecule from entering the pocket.

Having shown the competitive inhibitory effect of Bi(NO_3_)_3_ on Tet(X4) resistance enzymes, we next explored the modes of action of bismuth atom alone by molecular docking. Unexpectedly, we found that bismuth atom alone could not bind directly to the active center of Tet(X4) protein, whereas it targeted the other position with a binding energy of −2.25 kcal/mol. Molecular dynamics simulation was further used to investigate the underlying mechanisms. By monitoring the change of protein energy over time, we found that with the calculation of 6 ns, the energy of the whole system tended toward a stable state at the end (Fig. 7A), indicating that the bismuth atom has caused more obvious changes in the structure of the protein, and finally reached another stable state. Consistently, the root mean square deviation (RMSD) difference results showed that the overall structure of the protein fluctuated more obviously in the first 2 ns and reached equilibrium after 2 ns (Fig. 7B). The overall structure fluctuated slightly after 2 ns, which was consistent with the energy fluctuation value trend, suggesting that the bismuth atom caused the structure changes of Tet(X4) protein. The protein overlay diagram from 0 to 6 ns (Fig. 7C) further verified that the overall structure of Tet(X4) protein indeed changed in the presence of bismuth.

Next, we explored the impact of combined bismuth atoms on the protein binding pocket. In the absence of bismuth atom, D308 could form only a weak hydrogen bond with FAD (Fig. 7D). In contrast, in the presence of bismuth atom, D308 formed an extremely strong salt bridge with bismuth atoms and led to the drastic change in the amino acid area around D308 (Fig. 7E). Furthermore, we compared the structure of the protein binding pocket at 0 and 6 ns. Consistent with the energy and RMSD analysis, the binding pocket structure of Tet(X4) protein at 6 ns had a greater change than that at the 0 ns, and the binding pocket was shifted to inward, resulting in a reduction in the binding pocket space, which prevented the tigecycline from entering into the binding pocket (Fig. 7F to H). Together, our data show that, unlike Bi(NO_3_)_3_, the bismuth atom alone can bind to the Tet(X4) protein in a noncompetitive manner and alter the structure of binding the pocket, thereby antagonizing the enzymatic activity of resistance protein on tigecycline.

## DISCUSSION

The rapid evolution of antibiotic resistance has deepened the need for new antibacterial strategies. During the past decades, metal compounds have been widely used in biomedicine and other fields because of their unique and excellent antibacterial properties. However, the synergistic activity of metallo-agents with clinically relevant antibiotics is still not fully known. Bismuth, a group V metal, has antimicrobial properties (similar to silver). Some bismuth compounds have excellent biological activity, such as antileishmanial and anticancer properties (26). Their potential roles in responding to the drug resistance crisis have attracted increasing attention in recent years. Our study found that bismuth drugs, particularly Bi(NO_3_)_3_, had a remarkable synergistic effect with tigecycline in all tested *tet*(X)-positive bacteria rather than in *tet*(X)-negative bacteria, and subinhibitory concentrations of Bi(NO_3_)_3_ suppressed the resistance evolution of tigecycline-resistant gene *tet*(X). Effectiveness and safety are the two most important factors in the drug development. In fact, unlike other heavy metals, the toxicity of bismuth and its related compounds to the human body is negligible. Known as a “green metal” (23), many bismuth compounds are actually less toxic than sodium chloride (27). The reasons for its low toxicity in humans have begun to be understood. An exciting study showed that glutathione and multidrug resistance protein transporter could result in a self-propelled disposal of bismuth drugs in human cells but not in pathogens that lack glutathione (28). Nevertheless, more preclinical studies are warranted to verify the *in vivo* efficacy and safety of this drug combination.

In the mechanism studies, we found that five bismuth drugs exerted potent synergistic activity with tigecycline by inhibiting the activity of Tet(X4)-modifying enzyme. Interestingly, molecular docking and dynamics simulation analysis showed that Bi(NO_3_)_3_ and the bismuth atom had different mechanisms to inactivate Tet(X4). Specifically, Bi(NO_3_)_3_ inhibited Tet(X4) enzyme by competitively binding to the active center of Tet(X4) protein. In contrast, a single bismuth atom can also bind to the Tet(X4) protein, but in a noncompetitive manner, and further affected the structures of the binding pocket. This may be related to the unique characteristics of Bi(III); the larger ion radius makes it have strong deformability and polarization ability, which can overcome steric hindrance and accept multiple pairs of electrons to form complexes, easily coordinating with oxygen and nitrogen atoms (17). The differences in the mechanisms of individual Bi atom and Bi(NO_3_)_3_ indicated that NO_3_^−^ plays a critical role in the potentiation of Bi(NO_3_)_3_ to tigecycline. It also implied that Bi(NO_3_)_3_ may have dual modes of action to antagonize Tet(X4) enzymes in both competitive and noncompetitive pathways. Previous studies have shown that a series of proteins may be the potential targets of bismuth drugs, such as lactoferrin (29), metallothionein (30), and serum albumin (31). Additionally, proteomics technology was used to elucidate the mechanisms of bismuth drugs in killing Helicobacter pylori. It was found that bismuth metal acts on histidine and cysteine residues through noncovalent effects (32). As a recent report clarified, the synergistic activity of Bi(NO_3_)_3_ and meropenem found in our study maybe also be due to the substitution of Zn(II) ions by Bi(III) and cause the release of Zn(II) cofactor (20).

In summary, we reveal that bismuth compounds, especially bismuth nitrate, effectively potentiate the antibacterial activity of tigecycline against *tet*(X)-positive bacteria by inhibiting the enzymatic activity of Tet(X4). Meanwhile, the combined use of bismuth nitrate and tigecycline suppresses the evolution of Tet(X), thereby preventing the development of high levels of tigecycline resistance. To conclude, our study shows that bismuth drugs represent a class of novel and potent inhibitors of Tet(X) resistance enzymes, opening a new horizon for the treatment of recalcitrant infections caused by Tet(X)-positive pathogens together with tigecycline.

## MATERIALS AND METHODS

### Antibacterial tests.

According to CLSI 2018 guidelines (33), the MIC values were determined by microbroth dilution method. The drugs were twofold diluted in a 96-well microliter plate containing Mueller-Hinton broth (MHB), and then an equal volume (100 μL) of 1.5 × 10^6^ CFU bacterial suspension was added to each well. After that, plates were incubated at 37°C for 16 to 18 h. The lowest compound concentration without bacterial growth was recognized as the MIC.

### Checkerboard assays.

The synergism between antibiotics and bismuth compounds was measured using a 96-well flat-bottom plate according to previous studies (24, 34). A total of 100 μL MHB was added to each well to construct an 8 by 8 system. Antibiotics and bismuth compounds were twofold diluted in the plate and then mixed with equal volumes (100 μL) of bacterial suspension. After that, plates were incubated at 37°C for 16 to 18 h. Subsequently, a microplate reader was used to measure the optical density (OD) value at 600 nm. The synergism was determined by the FICI value, which was calculated by the following formula: FICI = FICI_A_ + FIC_B_ = MIC_AB_/MIC_A_ + MIC_BA_/MIC_B_. MIC_A_ and MIC_B_ are the MIC of A or B alone, respectively, MIC_AB_ is the MIC of A in the presence of B, and MIC_BA_ is the MIC of B in the presence of A. Synergism is defined when FICI is ≤0.5.

### MPC determination.

Briefly, 10^10^ CFU of E. coli B3-1 suspension was plated on LB agar plates containing different concentrations of tigecycline and Bi(NO_3_)_3_. After culturing the samples at 37°C for 72 h, we observed whether there were colonies growing on the plate. The MPC value was the lowest drug concentration that inhibits the growth of drug-resistant mutant clones. The experiment was performed with three biological replicates.

### Resistance development studies.

At subinhibitory concentrations of tigecycline or the combination of tigecycline and Bi(NO_3_)_3_, E. coli B3-1 and A. baumannii C222 were passaged continuously, respectively. The MIC of tigecycline was determined every four passages. According to the measured MIC, the drug concentrations for passage were increased, which were always maintained at subinhibitory levels. The passage lasted for 28 days.

### Tet(X) enzyme activity analysis.

The degradation of tigecycline by Tet(X4) enzyme and whether Bi(NO_3_)_3_ has the ability to inhibit enzyme activity were tested by the agar well diffusion method (4). The constructed BL21(DE3) + pBAD30-*tet*(X4) was inoculated into 1 mL LB broth. When the bacteria were grown to an OD value of 0.5 at 600 nm, 20 μM arabinose was added to induce Tet(X4) expression for 12 h. After centrifugation, the supernatant was separately inoculated with tigecycline (8 μg/mL) or its combination with different concentrations of Bi(NO_3_)_3_ (125, 250, 500, and 1,000 μg/mL) and cultured in a 37°C shaker for 12 h. Meanwhile, 100 μL of E. coli ATCC 25922 suspension was spread evenly on the Mueller-Hinton (MH) agar plate, a 6 mm-diameter sterile agar puncher was used to punch holes on the agar plate, and the bottom was sealed with a layer of hot agar. After centrifuging and filtering with a 0.22-μm filter, 30 μL coculture supernatant was added into the agar holes and incubated at 37°C for 18 h, and the diameter of each group of inhibition zones was measured and compared.

### Molecular docking and dynamics simulation.

Tet(X2) protein (2XYO, A chain) containing coenzyme FAD was used as a template. The homology modeling of Tet(X4) protein and template protein 2XYO was performed using SwissModel (35). Molecular docking between Bi(NO_3_)_3_ molecules, bismuth atom, and Tet(X4)-FAD complex system was carried using Autodock with random blind docking methods (36, 37). The related operations of molecular dynamics simulation were conducted using the Desmond module in the Schrodinger software package (38).
