# Supplementary material for: Bismuth Drugs Reverse Tet(X)-Conferred Tigecycline Resistance in Gram-Negative Bacteria
Source: Microbiol Spectr. 2022 Feb 9;10(1):e01578-21. doi: 10.1128/spectrum.01578-21 (PMC8826830; doi:10.1128/spectrum.01578-21)
Supplement: SUPPLEMENTAL FILE 1 — Supplemental material. Download SPECTRUM01578-21_Supp_1_seq4.pdf, PDF file, 0.3 MB [file spectrum01578-21_supp_1_seq4.pdf]

Supplementary Material

Figures

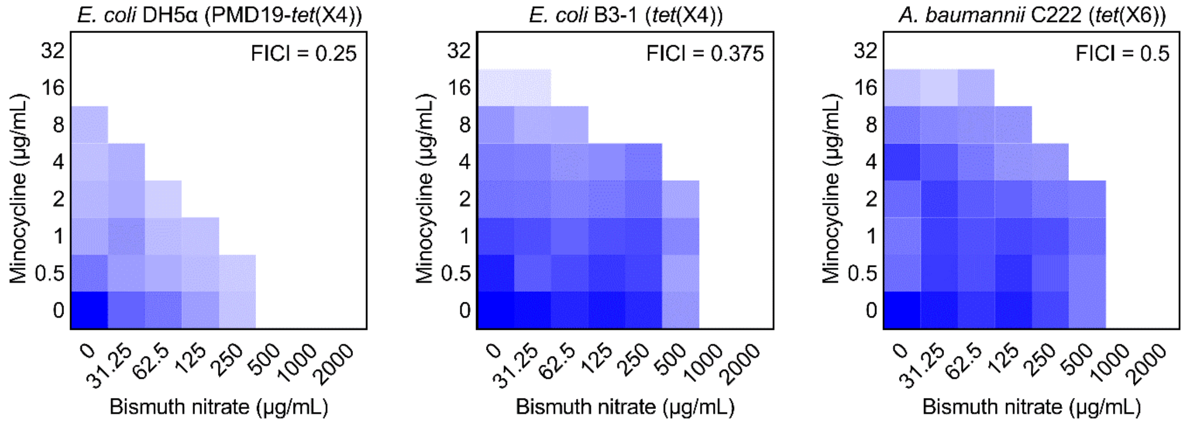

Figure S1 Synergistic activity of bismuth nitrate and minocycline against *tet*(X)-positive bacteria.

Dark blue regions represent higher cell density. Data represent the mean of two biological replicates.

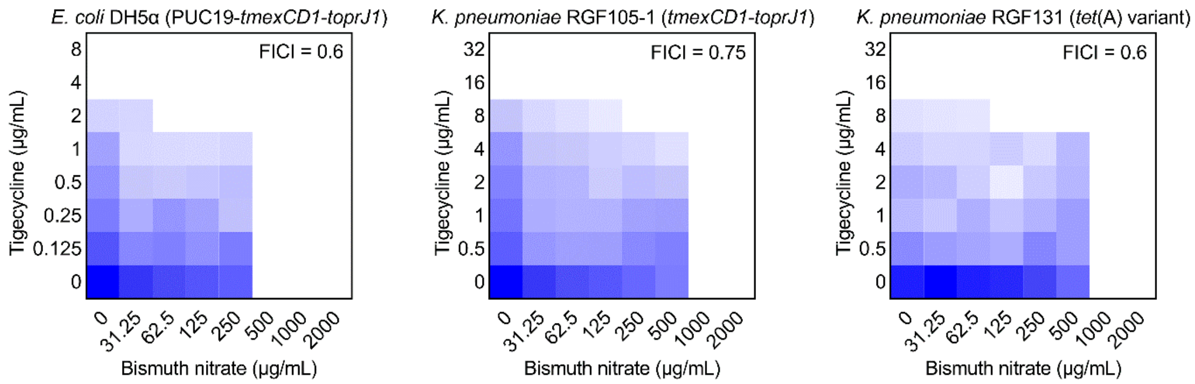

Figure S2 Checkerboard broth microdilution assay between bismuth nitrate and tigecycline against *tmexCD1-toprJ1*- and *tet*(A) variant-positive strains.

Dark blue regions represent higher cell density. Data represent the mean of two biological replicates.

Tables

Table S1 Synergistic activity of bismuth nitrate in combination with antibiotics of different mechanisms against *E. coli* B2 or *E. coli* B3-1.

Targets	Antibiotics	MIC[a] (μg/mL)	FIC index	MIC[b] (μg/mL)	Potentiation (fold)[c]
Cell wall	Ampicillin	>256	2	>256	--
	Meropenem	32	0.375	4	8
	Vancomycin	64	2	64	--
Protein	Doxycycline	16	2	16	--
	Tigecycline	32	0.125	2	16
RNA synthesis	Rifampicin	128	2	128	--
DNA synthesis	Ciprofloxacin	32	2	32	--
Cell membrane	Colistin	16	2	16	--

[a/b]MICs of antibiotics in the absence or presence of a quarter-MIC of bismuth nitrate.

[c]Degree of antibiotics potentiation in the presence of a quarter-MIC of bismut nitrate.

--, none of potentiation activity.

Table S2 Potentiation potency of bismuth nitrate with tigecycline against *tet*(X4)-positive clinical pathogens.

Pathogens	Source	MIC[a] (μg/mL)	MIC[b] (μg/mL)	FIC index	Potentiation (fold)[c]
E. coli B8-1	Blood	32	2	0.188	16
E. coli B9-1		4	0.5	0.25	8
E. coli W6-1	Water	16	1	0.188	16
E. coli W8-1		16	2	0.25	8
E. coli S2-1	Soil	32	2	0.125	16
E. coli S3-1		32	2	0.125	16
E. coli T29	Trunk	32	4	0.25	8
E. coli T18-1		16	4	0.375	4
E. coli P3-1	Pork	16	2	0.25	8
E. coli P12-1		16	2	0.25	8
E. coli F14	Feces	32	2	0.125	16
E. coli F12-1		32	2	0.188	16

[a/b]MICs of tigecycline in the absence or presence of a quarter-MIC of bismuth nitrate.
[c]Degree of tigecycline potentiation in the presence of a quarter-MIC of bismuth nitrate.

Table S3 Synergistic activity of bismuth compounds with tigecycline against *E. coli* DH5α (PUC19-*tet*(X4)).

Bismuth compounds	MIC[a] (μg/mL)	FIC index	MIC[b] (μg/mL)	Potentiation (fold)[c]
Bismuth sulfate	16	0.25	2	8
Bismuth subnitrate	16	0.25	2	8
Bismuth ammonium citrate	16	0.25	2	8
Bismuth thiflate	16	0.25	2	8

[a/b]MICs of tigecycline in the absence or presence of a quarter-MIC of bismuth compounds.
[c]Degree of tigecycline potentiation in the presence of a quarter-MIC of bismuth compounds.

Table S4 Potentiation of bismuth nitrate to tigecycline against Tet(X)-negative or positive Gram-negative pathogens.

Pathogens and genotypes	MIC[a] (μg/mL)	FIC index	MIC[b] (μg/mL)	Potentiation (fold)[c]
**Tet(X)-negative pathogens**				
E. coli ATCC 25922	0.125	2	0.125	--
S. enteritidis ATCC 13076	0.25	2	0.25	--
E. coli DH5α (PMD19)	0.25	2	0.25	--
**Tet(X)-positive pathogens**				
K. pneumoniae HS192 (*tet*(X4))	32	0.25	4	8
A. baumannii C222 (*tet*(X6))	32	0.375	4	8
E. coli DH5α (PMD19-*tet*(X4))	16	0.188	2	8

[a/b]MICs of tigecycline in the absence or presence of a quarter-MIC of bismuth nitrate.

[c]Degree of tigecycline potentiation in the presence of a quarter-MIC of bismut nitrate.

--, none of potentiation activity.
